# Impact of interaction networks of B cells with other cells on tumorigenesis, progression and response to immunotherapy of renal cell carcinoma: A review

**DOI:** 10.3389/fonc.2022.995519

**Published:** 2022-11-17

**Authors:** Yu-qi Wang, Wen-jin Chen, Wen-yan Li, Xiu-wu Pan, Xin−gang Cui

**Affiliations:** ^1^ Department of Urology, Xinhua Hospital, School of Medicine, Shanghai Jiaotong University, Shanghai, China; ^2^ Department of Urology, Third Affiliated Hospital of the Second Military Medical University, Shanghai, China

**Keywords:** renal cell carcinoma, tumor microenvironment, B cells, immune cells, interaction network, immunotherapy

## Abstract

Ample evidence indicates that the development and progression of renal cell carcinoma (RCC) are complex pathological processes involving interactions between tumor cells, immune cells and stromal components. Tumor infiltrated immune cells determine whether tumor advancement is promoted or inhibited. Among them, infiltrated B lymphocytes are present in all stages of RCC, playing a major role in determining tumor formation and advancement, as an essential part in the tumor microenvironment (TME). Although the advent of targeted and immune therapies has remarkably improved the survival of patients with advanced RCC, few cases can achieve complete response due to drug resistance. In this review article, we intend to summary the recent studies that outline the interaction networks of B cells with other cells, discuss the role of B cells in RCC development and progression, and assess their impact on RCC immunotherapy.

## Introduction

Renal cell carcinoma (RCC) is one of the most prevalent malignant tumors in the human urinary system, accounting for 2-3% of all tumors worldwide (1). Although considerable progress has been made in targeted therapies in improving the 5-year survival rate, the overall clinical outcomes remain unsatisfied due to postoperative recurrence, metastasis or chemotherapy resistance (2, 3). Recently, analysis of the tumor microenvironment (TME) has emerged as a potential approach of RCC treatment (4). Researchers have concentrated on tertiary lymphoid structures (TLS) and lymphoid aggregates in non-lymphoid tissues, where B cells as the principal components in surrounding T cell zones interact with other cells. TLS appear in various stages of maturity in different tumor types, culminating in germinal center (GC) formation (5). Tumor infiltrated B cells are mostly associated with TLS compared with other immune cells. Although B cells can also be recruited to the tumor bed directly rather *via* TLS, the density of B cells is relatively low under these circumstances. B cells and tumor-associated TLS can be found in the TME of most cancer types and are correlated with tumor immunotherapies (5).

The TME provides a complex tumor ecosystem composed of cancer cells and multiple non-cancerous cells, playing decisive roles in tumor initiation, progression and dissemination (6, 7). Cancer cells interact with stromal cells and immune cells, working together rather separately to form the principal structure of the TME. As one of the characteristics of cancer, prolonged inflammation initiates tumorigenesis or supports tumor progression, during which the entire immune landscape is altered drastically (8). To progress in the body, tumors have derived many mechanisms to escape immune surveillance by producing neoantigens to interfere with the immune system. Therapies targeting the TME have been studied and implemented from bench to bedside. It is described that the therapy of PD-1 and PD-L1 blockade has made therapeutic improvements about metastatic tumors in some reviews, but the objective response rates still remain unsatisfactory. Thus, it is worthwhile to investigate the multiple associations in TME to further explore B lymphocyte-targeted therapies of RCC.

**Figure 1:**
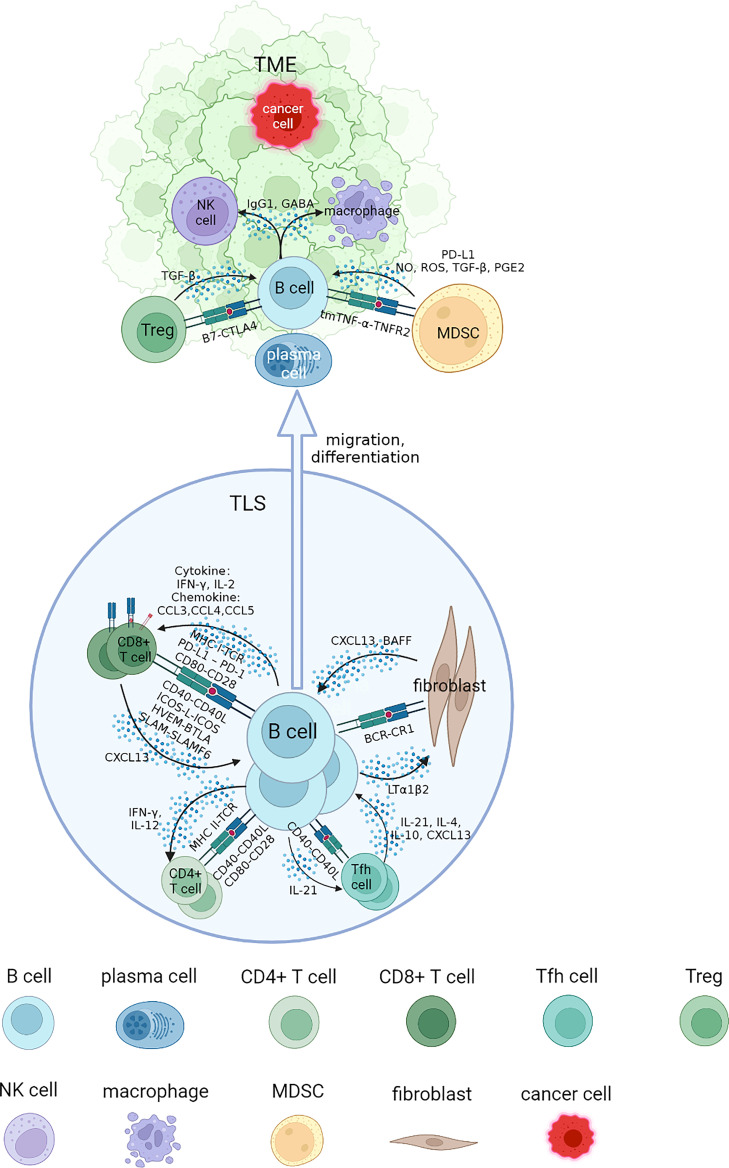
Interaction networks between B cells and other cells in tertiary lymphoid structure (TLS) and tumor microenvironment (TME). B cells cooperate with other cells to perform an immunomodulatory role on tumorigenesis and progression. B cells could be attracted to TLS by CXCL13, where they mature and interact with different types of T cells through specific co-stimulatory and co-inhibitory signal pairs such as CD40-CD40L, CD80-CD28. The class switch recombination and differentiation of B cells are promoted by cytokines including IL-4 and IL-10, and B cells receive IL-21 to develop into plasma cells and memory B cells, migrating to tumor bed. They cooperate with NK cells and macrophages to implement ADCC and ADCP process through antibodies including IgG1 in TME. Some inhibit regulatory effects of MDSCs and Tregs on B cells in TME could promote tumor progression. NK, natural killer; MDSC, myeloid-derived suppressor cell; NO, nitric oxide; ROS, reactive oxygen species; PGE2, prostaglandin E2.

In this review, we address questions regarding the interaction networks of B cells and other cell types by focusing on the association of infiltrated B cells with tumorigenesis, progression and response to immunotherapy of RCC. These cells are collaboratively engaged in aspects of the tumor process and immune TME. We will elucidate each part of B cell interaction that affects immune response in RCC from four specific sections, and review the advances of B cells and TLS with tumor immunotherapies (**Figure 1**).

## The interaction of B cells with T cells

B lymphocytes first differentiate from hematopoietic stem cells in the bone marrow, experiencing continuous development into immature B cells, and migration to second lymphoid tissues, where they mature (9). The process in the bone marrow involves immunoglobulin light chain gene rearrangement, and VDJ gene segment recombination. Upon stimulation by the antigen, B cells experience antibody class switching and somatic hypermutation in the GC, which has proved to be the mechanism for affinity maturation of antibodies (10, 11). The initiation of GC reaction involves several distinct cell types *via* a coordinated cascade, guiding antigen-engaged B cells into GC reaction. These processes along with GC formation are well assisted by other immune cells, especially T cells. Additionally, B cells are one of the critical components of the humoral immune system, regulated by numerous control mechanisms at both cellular and molecular levels. The induction of antibody response also requires the collaboration of T and B cells.

The function that T cells provide assistance for B cells has been recognized for decades, resulting in the demonstration of thymus-derived helper cells (12–14). This two-lymphocyte lineage model indicates issues of synergistic function and their cooperation. As the close partner, their history has been reviewed in detail (15). For various types of cancers, the associated T and B networks have already generated interests in immune therapy in many recent studies, and research highlighted the emerging roles of B cells in tumor immunity and the focus on T cell response. These findings could guide a protocol and provide potential therapeutic strategies for RCC patients.

### B cells with Tfh cells

T follicular helper (Tfh) cells are the crucial partner of infiltrated B cells, whose crosstalk within TLS in the TME has been verified to affect tumor immunotherapy (16). There exists a bidirectional interaction in the Tfh-B combination. B cells are essential for Tfh cell formation by mediating PI3K-dependent migration of CD4+T cells into follicles (17). In a murine model of lung adenocarcinoma, B cells were found to promote tumor-specific CD4+Tfh cell differentiation, which then produced IL-21 to enhance CD8+T cell responses to drive anti-tumor immunity (18). In turn, mature Tfh cells provide binding CD40 ligand (CD40L or CD154) and IL-21 for GC B cell differentiation into memory B cells and plasma cells (19–21). IL-21 also plays a pivotal role in Tfh cell formation, B cell growth and class switch recombination (CSR), and maintains the expression of Bcl-6 in GC B repertoires (20, 22, 23). These processes are modulated by other Tfh cell cytokines, including IL-4 and IL-10, which favor IgG and IgA production, respectively (17). In addition to offering help for T-dependent B cells, some specialized CXCL13-producing Tfh cells could guide CXCR5+ lymphocyte migration, promote local memory B cell differentiation, and behave as a potential surrogate marker for GC reaction and TLS formation (24–26). Studies suggested that anti-PD-1 therapy in cancer could strengthen B cell capacity by increasing circulating Tfh cells (27). An investigation of the clinical data of clear cell RCC (ccRCC) showed that immune-related prognostic gene signatures were differentially expressed in distinct lymphocyte clusters (28). In a study of metastatic ccRCC patients under ICB therapy, unswitched memory B cells correlated positively with Tfh cells, TLS and CD20+ B cells, associated with higher response rate and better overall survival (29). The related genes and the interaction mechanisms of B cells and Tfh cells may prove to be a biomarker for assessing prognosis of RCC and screening precise patients for specific immunotherapies.

### B cells with CD8+T cells

#### Effects of B cells on CD8+T cells

Some evidence proves that B cells can exert a direct or indirect effect on CD8+T cells. B cells present tumor-specific antigens that they have captured by B-cell receptors (BCRs) to CD8+T cells (30), release cytokines, and participate in the formation of co-stimulatory and co-inhibitory receptors (31). Some cytokines such as IL-2 and IFN-γ secreted by B cells can bind to the receptors on CD8+T cells (31). The co-stimulatory and co-inhibitory signal pairs include CD80-CD28, CD40-CD40L, ICOS-L-ICOS; PD-L1-PD-1, HVEM-BTLA, SLAM-SLAMF6 (32). In addition, B cells can indirectly support CD8+T cells by interacting with Tfh cells through CD40-CD40L (32). *In vitro*, B cells were found differentiating into plasmablast-like cells and expressing T cell recruitment chemokines like CCL3, CCL4 and CCL5. Plasmablast-like cells increased the activation of PD-1+T cells *via* anti-PD-1 blockade, and their frequency could predict response and survival to immune checkpoint inhibitor (ICB) (33). In a metastatic RCC pre-surgical trial, B cell signatures were found enriched in tumors of responders of ICB treatment, which was also confirmed in another ICB-treated cohort of melanoma patients (34). Accordingly, we speculate that B cell subpopulations within the TLS could modulate T cell antitumor response and serve as a possible ICB response predictor of RCC.

#### Effects of CD8+T cells on B cells

Current reports suggest that T cells may appear first in the tumor sites and then promote the recruitment of B cells. CD8+T cells activated by TGF-β will upregulate CD103 and release CXCL13, a potent B cell chemoattractant that binds to CXCR5 receptor of B cells, and then mediate B cell recruitment and TLS formation (35). Researchers observed similar phenomenon that infiltrated B cells were prone to colocalize with CD8+T cells (36), and the significant correlations between them imply their cooperation in a tumor-killing effect of several malignancies (31). A study on ccRCC demonstrated that the abundance of intratumoral CD8+T cells secreting CXCL13 was associated with increased TLS and immunoevasive TME, functioning as a potential immunotherapeutic marker for ccRCC treatment (37). Another study illustrated the prognostic value of CCL4, CCL5, CCL8, CCL19 and CXCL13 expression in ccRCC. Besides, DNA deletion of TLS gene signatures could greatly indicate poor outcome in ccRCC patients compared with wild-type gene signature (38). All these results provide insights into how B cells cooperate with CD8+T cells and their roles in ICB treatment, which may assist the development of RCC therapeutic targets.

### Roles of Tregs on B cells and plasma cells

#### Direct regulations

Regulatory T cells (Tregs) dampen B cell proliferation and plasma cell formation (39). Hyung et al. reported that Tregs could directly interact with B cells to suppress immunoglobin (Ig) response, production and CSR without the help of Tfh cells, and downregulate relative gene expressions of naïve B cells *via* contact-dependent mechanisms (40, 41). Interestingly, it was elucidated that Tregs reduced all other Ig production but induced B cells to produce IgG4 in a cell contact-, TGF-β- and IL-10-dependent manner (42, 43). Studies have demonstrated that Tregs are related to negative outcomes in RCC patients (44), and the expansion of Tregs could limit the function of IL-2 in RCC treatment (45).

#### Indirect regulations

Tregs downregulate the function of T helper (Th) cells through various mechanisms, resulting in adverse effects on B cells (46). Tregs could also suppress *in vitro* B cell Ig production by inhibiting Th function *via* TGF-β (47). In addition, Tregs migrate to follicles in the GC and regulate GC B cell responses (48). A population of follicular regulatory T (Tfr) cells is also described, which share phenotypes of both Tregs and Tfh recruited during GC reaction, yet are distinct from both (49, 50). Tfr cells could balance the Tfh-mediated B cell responses through CTLA-4 expression (17), and it has been proved that a lack of Tfr expressing CXCR5 and Bcl-6 could lead to greater GC reaction, associated with GC B cells, affinity maturation of antibodies and plasma cells differentiation (51). Consequently, the regulation of B cells in the GC reaction is well counterbalanced by Tfh and Tfr cells. A recently published study indicated another population of Foxp3+T cells which existed in the end-stage of GC, displaying an immediate phenotype of Tfh and Tfr cells (52). Collectively, these suggest the indirect effects of Tregs on B and plasma cells, mainly through Tfh and Tfr cells in the GC.

## B cells cooperate with NK and macrophages

### ADCC and ADCP

The antitumor activity of NK cells depends on their antibody-dependent cell-mediated cytotoxicity (ADCC) when they encounter tumor-specific IgG1 antibodies secreted by plasma cells (30, 53). Unfortunately, some tumor-derived IgG may impede the ADCC process by binding with specific antigens lacking complement activation. To against high PD-L1-expressing tumors, NK cells combining with anti-PD-L1 antibodies helps promote ADCC activity (54). However, NK cells are generally scarce and become anergic in the TME (55). Tumor-associated macrophages (TAM) participate in the ADCC or phagocytosis process *via* antibody-dependent cell-mediated phagocytosis (ADCP). Intriguingly, Su et al. reported an unexpected finding that ADCP macrophages may inhibit ADCC and T cell-mediated cytotoxicity by upregulating immune checkpoint such as PD-L1 and indoleamine 2,3-dioxygenase to cause immunosuppression (56). Therefore, a combination of therapeutic antibodies and ICB potentially provide synergistic effects in RCC treatment.

### B cells with macrophages

Fc gamma receptor (FcRγ), a receptor binding to Fc region of an antibody, could modulate protumor bioactivities of macrophages. In the absence of B cells or FcRγ, macrophages were found reprogramming towards the M1-type inflammatory state (57). Specifically, B cells could foster tumor development through FcRγ by activating pro-angiogenesis and tissue remodeling of myeloid cells, especially macrophages and mast cells (57). Besides, the reprogramming of macrophages also regulates CD8+T cell recruitment. Researchers discovered that by using B cell-depleting αCD20 monoclonal antibody as an anticancer monotherapy in mice, the chemokine expression of macrophages altered, thus improving CD8+lymphocyte infiltration and chemotherapy response in squamous cell carcinoma (58). A study of pancreatic ductal adenocarcinoma showed that Bregs could induce polarization of M2 macrophages and aggregation of Tregs, thus resulting in T cell suppression (59). Likewise, in ccRCC cohorts, researchers observed that compared with early stage tumors, pro-inflammatory macrophages were reduced, while suppressive M2-like macrophages were elevated in advanced disease (60). In conclusion, immunosuppressive B cells and plasma cells tend to facilitate TAM conversion to protumoral M2 phenotype, while effector B cells could promote TAM conversion to tumoricidal M1 phenotype (30). In the study of ccRCC, researchers found that TAM-derived chemokine CCL5 displayed a correlation with increased B cells and CD8+T cells, and decreased CD4+T cells. Elevated CCL5+TAMs infiltration exhibited higher tumor-infiltrated lymphocytes, but reduced TLS, correlated with a distant prognosis of ccRCC patients (61).

Although interactions of lymphocytes are regulated with various cell-bound proteins, small metabolites, as essential intermediates in biochemical processes, could reflect neighboring cells when released into extracellular milieu (62, 63). Metabolite and neurotransmitter GABA, synthesized and secreted by activated B cells and plasma cells, could promote monocyte differentiation into macrophages, polarizing towards an anti-inflammatory phenotype. They function as protumor cells through releasing interleukin (IL)-10 and limiting CD8+T cell function (64). Furthermore, in RCC microenvironment where B cells and IgA+ plasma cells were highly infiltrated, GABA was almost exclusively detected. It may suggest that GABA could regulate T cells and monocytes in the TME of RCC (64). Further studies are needed to unravel the occasions of intracellular metabolites mediate interactions between cells to inhibit tumor growth or enhance B cell immunity to cancer.

## The interaction between fibroblasts, dendritic cells and B cells

Fibroblast reticular cells (FRCs) are considered immunologically specialized myofibroblasts originating from mesenchymal stem cells (65). Inside the encapsulated sponge-like network of FRCs in lymph node congregated B cells, T cells, plasma cells, dendritic cells (DCs) and macrophages. FRCs in B cell zone provide B cell growth factor—B cell-activating factor (BAFF) and transcribe B cell chemoattractant CXCL13 to maintain and attract B cells in support of B cell survival (66). During infection, FRCs rapidly upregulate the CXCL13 expression *via* crosstalk with B cells, and control the expansion of B cell follicle boundaries upon inflammation (67).

Of note, DCs are specialized non-hematopoietic stromal cells residing in lymphoid follicles and GC. They participate in optimal selection of B cells that secret antibodies (68). In addition, B cells populate in the network of follicular dendritic cells (FDCs). Mature TLS comprise a GC with CD23+FDCs, which could present antigens to B cell selection with high-affinity BCRs, and promote B cell activation and differentiation (69). FDCs present naïve antigens to GC B cells *via* complement receptors 1 (CR1) (70). In cohorts of prospective and retrospective lung cancers, a high density of mature DC is strongly linked with a substantial infiltration of T cells with effector-memory characteristics, T-cell activation gene expression and T-helper 1 phenotype (71). These results indicate that mature DCs may generate some specific immune contexture that influences infiltrated B cells and TLS in TME.

Researchers have demonstrated that fibroblasts could directly support TLS formation and development (72). In the settings of inflammation and persistent antigen presentation, TLS-associated fibroblasts differentiate from locally activated mesenchyme (73). Likewise, stromal cell priming and lymphocytes accumulation have close relationships and the former might occur before lymphocyte migration. This kind of cancer-associated fibroblasts (CAFs) play a pivotal role as lymphoid tissue organizer cells (LTo) and coordinate with multiple cell types that synergistically act as lymphoid tissue inducer cells (LTi) cells, such as Intratumoral CD8+T cells and B cells that drive TLS development. CAFs work as surrogate LTo and participate in TLS formation and orchestration. On the other hand, the reticular network of CAF is mediated by CD8+T cells, and its accumulation relies on the recruitment of B cells expressing lymphotoxin, namely intratumoral LTα1β2+B cells. Imaging analysis has confirmed an elevated density of B cells coexisting with a reticular network of LTo-like CAF (74). Analysis of TCGA data of RCC showed that CAF infiltration was higher in RCC, especially in RCC with advanced tumor pathological grades and stages, than that in normal tissues (75). In addition, studies of RCC have revealed that TLS are sites of *in situ* B cell maturation into plasma cells. The plasma cells formed in the TLS are embedded in the dense network of fibroblasts, and disseminate into the tumor beds along fibroblastic tracks (76).

## MDSC-mediated regulation of B cell response

Myeloid-derived suppressor cells (MDSCs) were first termed in 2007, representing a series of immunosuppressive macrophages, DCs and granulocyte precursor cells produced in response to tumor-derived cytokines (77). Since then, MDSCs have been considered a great obstacle for cancer immunotherapies because they have close relationships with other immune cells. Studies have identified that normal B cells could be transformed into a subtype of immune regulatory B cells (Bregs) inhibiting T cell response in the presence of MDSCs. Besides, some immune checkpoint molecules including PD-1 and PD-L1 might be changed and remolded predominantly. A novel MDSC-Induced B cell subset (PD-1-PD-L1+CD19+) has been demonstrated to inhibit T cell responses (78). Specifically, activation of the PI3K/AKT/NF-kB axis enhances the PD-1-PD-L1+ Breg protumor function (79). Another study on glioblastoma discovered that MDSCs delivered functional membrane-bound PD-L1 *via* microvesicles to Bregs, conferring the effector phenotype and function (80). Cell experiments have elucidated that 1) MDSCs suppress B cell proliferation by releasing suppressive mediators; 2) MDSCs induce decreased expression of B cell surface markers including IgM, HLA-DR, CD80 and CD86; 3) MDSCs induce specific B cell subset phenotypic alterations including antibody-secreting cells death; and 4) MDSCs elicit specific gene transcriptional changes which are associated with apoptosis, class-switch regulation and B cell differentiation and effector function (81).

In addition, some indirect regulatory effects of MDSCs on B cells have also been elaborated. MDSCs increase the number of FoxP3+ Treg cells, and facilitate the development of Tregs (82, 83). Tregs, along with Bregs, have similar suppressive characteristics and close relationships with B cells. FoxP3+Treg cells inhibit antibody production, activation and differentiation of B cells. Besides, it is reported that MDSCs modulate B cells *via* different pathways, including TNF, STAT3 and TGF-signaling, and MDSC-derived nitric oxide (NO), reactive oxygen species (ROS), TGF-β, and prostaglandin E2 (PGE2) play roles in suppressing B cells (84). RCC is regarded as an immunogenic tumor, which elicits the influx of immune-inhibitory cells such as Tregs and MDSCs into the TME, resulting in immune dysfunction (85). Many possible mechanisms have been proposed to explain how MDSCs target immune suppression (86), and it has resulted in clinical response in some patients with RCC (85, 87). The regulation of B cells by MDSCs may be a prospective target for immunotherapy in RCC.

## Indications of TLS and immunotherapy

TLS have been identified in various types of tumors at every stage of disease, but their presence is in high heterogeneity between different cancer types and patients (88, 89). It is thought that TLS actively modulate antitumor immune activities rather than simply being a surrogate marker of rapid immune response (34). Mature TLS show indications of GC development. In colorectal cancer, TLS are associated with favorable outcomes and harbor prognostic information of disease recurrence (90). In lung squamous cell carcinoma, TLS are the independent prognostic marker and their development can be affected by chemotherapy and steroids (91). Interestingly, between different types of genitourinary cancer, one study showed that TLS displayed a distinct status in terms of the clinical outcome and immunogenomic profile (92). Researchers showed that the TLS detected in RCC cohort were less mature, contributing to poor outcomes, while in bladder cancer cohort, TLS were more mature with GC structures and associated with better outcomes (92). Another study certificated that in three gradual levels of immune infiltration of ccRCC clusters, higher abundance of T cells and TLS, suggesting an immune-enriched TME, was related to poor clinical outcome (93). These results suggest the heterogeneity of TLS and indicate that comparison of the TLS characteristics may help show differences in the immune TME and prognostic effects in RCC.

## B cells and immunotherapy

Recent studies have contributed to an appreciation for B cells influencing immunotherapy outcome (30). Researchers have already reached consensus on the surface phenotype markers of various B subtypes except Bregs (94), and single-cell RNA sequencing (scRNA-seq) technique provides a broader perspective on cell markers and characteristics. scRNA-seq and cell-cell communication analysis in several recent studies have demonstrated that interactions of infiltrated B cells could influence tumor clinical outcomes (**Table 1**). The heterogeneity across B-cell subpopulations has been studied by single-cell techniques. Some single cell methods have helped dissect tumor heterogeneity and study the anti-tumor drug responses. However, the specific B-cell gene signatures between different cancer types still need more investigations. When initiating an antitumor response, tumor-infiltrated B cells (TIL-Bs) are first required to be recognized by tumor-specific neoantigens *via* BCRs. Studies have suggested that complementarity determining region-3 (CDR3), highly changeable regions in the BCR, have the potential to be prognostic biomarkers of different malignancies (104–106). Furthermore, the diversity of intratumoral BCR repertoires could reflect clonal expansion in response to tumor-associated antigens. Compared to scRNA-seq, repertoire studies may better characterize TIL-Bs including the investigation of B-cell phenotypes and BCR diversity within the RCC microenvironment (107). Some distinct RCC-associated gene mutations displayed by genomic techniques also have correlations with BCR repertories. Researchers found that among RCC patients with mutations in KDM5C, PBRM1, VHL and PTEN, BCR repertoire diversity was decreased (108). Intriguingly, PBRM1 mutation is pertinent with immune checkpoint inhibiters (ICI) response of RCC (109, 110). Some gene segments may be enriched in TIL-Bs with particular gene expression phenotypes. Besides, some BCR pathway molecules are upregulated and BCR-related kinases play a role in the TME of various tumors, suggesting an anticancer activity of targeting BCR-immune complex and BCR-related kinases (106, 111). Overall, this may provide a new insight of exploring B cell subpopulations most affected by molecular features of tumor and contribute to new targets of immunotherapy with the combination of scRNA-seq and BCR repertories.

**Table 1:** Current research about the interactions of B cells influencing tumor clinical outcomes, using scRNA-seq or cell-cell communication analysis.

Tumor type	Interactions	Outcomes	Reference
**Non-small cell lung cancer (NSCLC), malignant pleural effusion (MPE)**	In MPE, Bregs interact primarily with CD4+ T cells (including Th1/17, Treg and Tfh), but not CD8+ T cells.	Bregs in MPE show great cell proliferation signaling and are related to poor clinical outcomes.	(95)
**Colorectal Cancer (CRC)**	Compared to non-tumor tissues - Enhanced interactions between non-immune cells (including MKI67+ goblet cells, DEFA5+DEFA6+ metaplastic paneth cells, colonocytes, and fibroblasts) and immune cells (including B cells, T cells and myeloid cells) in tumor tissues. - Altered interaction pathways between B cells and T cells.	- B, plasma and non-immune cells in tumor tissues exert important roles in shaping tumor microenvironment. - Proliferative B-cell signatures are enriched in tumors that respond to immunotherapy.	(96)
**CRC**	Compared to non-tumor tissues - Enhanced interactions between myeloid cells and lymphocytes (including B cells, plasma cells and T cells) in tumor tissue. - Enhanced interactions between B cells and T cells through CD48-CD244. - B cells tend to interact with SIGLEC10+ T cells and inhibit T cell activation. - Enhanced interactions between IgA+ IGLC2+ plasma cells and multiple types of T cells. - CCL8+ IGLC2+ plasma cells and cycling B cells interacte with CCR5+ T cells in CRC and recruit CCR5+ T cells to the tumor foci. - Attenuated interactions between epithelial cells and B cells, but the SIRPA-CD47 and NRG1-ERBB3 pathways are enhanced. These are associated with immune escape and epithelial-mesenchymal transition (EMT)-associated metastasis. Compared to early stage tumor tissue - Enhanced interaction of B cells with other immune cells in advanced tumor tissue. - IgA+IGLC2+ plasma cells, which are associated with poor prognosis, have significant interactions with myeloid cells and cytotoxic T cells.	- B cells and myeloid cells are predominantly responsible for immunoregulatory functions in CRC rather than CD4+ regulatory T cells. - B cells in early CRC tumors exhibit pre-B like tumor suppressors, while B cells in advanced CRC tumors tend to develop into plasma cells. - B cells in CRC may contribute to tumor progression.	(97)
**CRC**	B cells, MKI67+ T cells and dysfunctional T cells, interact with tumor-associated macrophages (TAMs), which are enriched by preoperative chemotherapy, through HLA-F-LILRB2 and HLA-DPB1-NRG1 pathways in the cell niche of primary tumors.	- Less-activated B cells are more prevalent in the tumor microenvironment of treatment-naïve tumors. - B cell activation is observed in tumors treated with preoperative chemotherapy.	(98)
**Nasopharyngeal Carcinoma (NPC)**	- The three exhausted T cell populations in TME (HAVCR2+, TOX+, and LAG3+ T cells) preferentially interact with memory B cells, innate-like B cells, unactivated B cells, and IFN-induced B cells, but not with plasma cells, naive B cells, and double-negative B cells. - Among them, B cells interact with HAVCR2+ and TOX+ exhausted T cell populations mainly through the CXCL13-CXCR5 axis.	- A higher abundance of B cells is correlated with a better clinical prognosis in NPC patients. - A higher percentage of double-negative B cells is predictive of worse survival rate in NPC patients.	(99)
**Esophageal squamous cell carcinoma (ESCC)**	Attenuated interactions between tumor cells and B cells compared to interactions between tumor cells and other immune cells in TME.	The specific cellular communication potentially shapes the unique TME in ESCC.	(100)
**Ovarian Cancer**	- Tumor-infiltrating B cells (B-TILs) interact with CD4+CXCL13 T cells as well as dysfunctional CD8+GZMB T cells through CXCR5-CXCL13, which is a possible mechanism for recruiting B cells into the tumor microenvironment. - B-TILs interact with endothelial cells *via* CCR7-CCL21, suggesting another possible mechanism for recruiting B cells into the tumor microenvironment.	Stromal cells and T cells participate in the recruitment of B cells in tumor and stromal compartments of ovarian cancer.	(101)
**Lymphoma**	- Malignant B cells receive suppressive signals from all four major T cell subsets (T helper, T toxic, Tfh, Treg) through CD80/CD86-CD28 and CD80/CD86-CTLA. - Malignant B cells interact with T helper cells and Tregs through BCMA-BAFF, BAFF-R-BAFF, and CD40-CD40LG. - Malignant B cells interact with Tfh cells through IL4-IL4R, IL4-IL13RA1 and IL21-IL21R.	- B cells modulate the frequency of various lymphoma-infiltrated T cell subsets to shape the microenvironment. - Malignant B cells are heterogenous among lymphoma patients with different proliferative activity. This is associated with lymphoma-specific transcriptional features.	(102)
**Head and neck squamous cell carcinoma (HNSCC), caused by environmental carcinogens or human papilloma virus (HPV)**	- B cells interact with CD4+ T cells in both HPV- and HPV+ TME. Besides, the spatial distance between B cells and CD4+ T cells is closer, which reflects the probability of interaction. - Interactions between GC B cells and TFH are only in HPV+ TME.	B cells in germinal center are observed in HPV+ tumor microenvironment, and correlate positively with the overall survival in HNSCC.	(103)

Antibodies produced by B cells have associations with effective antitumor immune response. Researchers demonstrated a high level of plasmablasts in the blood of metastatic RCC patients who had not exhibited tumor progression for over a year, and reactive antibodies from B cell response are commonly detected, which exhibit a great level of somatic hypermutation (112). A study indicated that TIL-Bs had unique antibody repertoire characteristics, including elevated clonal polarization and high somatic hypermutation rates in treated-tumor-bearing mice (113). The signs of B cell activation and clonal expansion were similarly discovered in other human malignancies (111, 114). These results suggest a possible persistent B cell response targeting tumor antigens. The identification of antibody repertoire signatures could perform as markers to identify tumor-reactive B cells, and provide a new paradigm for discovering antitumor antibodies with RCC diagnosis and immunotherapy.

## Can B cells and TLS be predictive and prognostic factors in RCC?

We have reviewed the crosstalk between B cells and other cells in the TME, highly expecting that indications of B cells and TLS with immunotherapy could be the predictive and prognostic factor in RCC. Some current studies and clinical trials have confirmed the value for different B subsets in RCC. A clinical study on the plasma sample of RCC patients demonstrated that the blood concentration of unswitched memory B cells was correlated with the response condition to immune checkpoint blockade and survival rate in metastatic ccRCC patients (29). Similarly, researchers found that a subset of B cells with a memory phenotype was associated with positive outcomes in RCC patients treated with immune checkpoint inhibitors, and could predict response to checkpoint immunotherapy (115). Meylan et al. (76) used spatial transcriptomics to investigate B cell immunity within intratumoral TLS in RCC, and proved that TLS sustained B cell maturation and antibody generation, with response to immunotherapy possibly *via* direct antitumor effects (76). It has been proved that RCC exhibits distinct immune phenotypes and proteogenomic characteristics (116). Growing evidence indicates the diversity and heterogeneity of TME and tumor cells affect immunotherapy (117). Although no large comparative study has been reported to explore the specific effects and mechanisms of B cells on RCC patients in clinical trials, the clinically related outcomes of B cells and TLS with RCC occurred in previous studies. Therefore, further investigations are needed to confirm the predictive and prognostic value of B cells and TLS in RCC.

## Conclusion

Increasing evidence has demonstrated the important role of B cells in tumor immunotherapy, and some innovative techniques including scRNA-seq and BCR repertoires have provided intensive insights into B cells and TME. In this review, we focus on the interaction networks of B cells with other cells in RCC microenvironment. Some subtypes of T cells including CD8+T cells and Tfh cells contribute to the recruitment, maturation and differentiation of B cells. Besides, B cells also act as a provider of antigen presenting cells and release cytokines to help T cells perform their duties in tumor sites. On the contrary, subsets of Tregs and MDSCs suppress the performance of B cells. In addition, NK cells and macrophages perform ADCC and ADCP function through using antibodies, and fibroblasts perform an essential role in the maturation of B cells in TLS. We speculate that the study of the interaction networks of immune cells can provide valuable information for RCC treatment, and how to improve the response rate to immunotherapy of RCC is an important issue that needs to be considered seriously.

## Author contributions

All authors had full access to all the data in the study and take responsibility for the integrity of the literature. All authors were involved in critical revision of the manuscript for important intellectual content. All authors contributed to the article and approved the submitted version.

## Funding

This work was sponsored by the National Natural Science Foundation of China (No. 81974391, 82072806, 82173265); the Clinical Research Plan of SHDC (SHDC2020CR4025); Shanghai “Rising Stars of Medical Talent” Youth Development Program: Youth Medical Talents - Specialist Program (X. Pan); Shanghai Municipal Commission of Health and Family Planning (20204Y0042); the Hospital Funded Clinical Research of Xinhua Hospital Affiliated to Shanghai Jiaotong University School of Medicine (21XHDB06).

## Conflict of interest

The authors declare that the research was conducted in the absence of any commercial or financial relationships that could be construed as a potential conflict of interest.

## Publisher’s note

All claims expressed in this article are solely those of the authors and do not necessarily represent those of their affiliated organizations, or those of the publisher, the editors and the reviewers. Any product that may be evaluated in this article, or claim that may be made by its manufacturer, is not guaranteed or endorsed by the publisher.
